# Supplementary material for: Production of an Anise- and Woodruff-like Aroma by Monokaryotic Strains of Pleurotus sapidus Grown on Citrus Side Streams
Source: Molecules. 2022 Jan 19;27(3):651. doi: 10.3390/molecules27030651 (PMC8838675; doi:10.3390/molecules27030651)
Supplement: Supplementary file 1 [file molecules-27-00651-s001.zip › molecules-1556546-supplementary.pdf]

Article

Production of an Anise- and Woodruff-like Aroma by Monokaryotic Strains of *Pleurotus sapidus* Grown on *Citrus* Side Streams

Friederike Bürger [1], Maximilian Koch [1], Marco A. Fraatz [1,2], Alejandra B. Omarini [3,4], Ralf G. Berger [4] and Holger Zorn [1,2,*]

[1] Institute of Food Chemistry and Food Biotechnology, Justus Liebig University Giessen, Heinrich-Buff-Ring 17, 35392 Giessen, Germany; friederike.buerger@lcb.chemie.uni-giessen.de (F.B.); maximilian.koch@lc.chemie.uni-giessen.de (M.K.); marco.fraatz@lcb.chemie.uni-giessen.de (M.A.F.); holger.zorn@uni-giessen.de (H.Z.)
[2] Fraunhofer Institute for Molecular Biology and Applied Ecology, Ohlebergsweg 12, 35392 Giessen, Germany
[3] CONICET Asociación para el Desarrollo de Villa Elisa y Zona Héctor de Elia 1247, E3265 Villa Elisa (Entre Ríos), Argentina; alejandra.omarini@adesarrollo.com.ar
[4] Institute of Food Chemistry, Leibniz University Hannover, Callinstrasse 5, 30167 Hannover, Germany; rg.berger@lci.uni-hannover.de
* Correspondence: holger.zorn@uni-giessen.de; Tel.: +49-(0)-641-99-349-00

Supplementary Material

Table S1: Classification of PSA strains into odor categories after surface cultivation on *Citrus*-supplemented medium; 1: musty, unpleasant, 2: PSA-typical, but weak and partly with off-notes, 3: PSA-typical, 4: woodruff-like, coumarin, herbaceous and partly sweetish, Dk: dikaryon, Mk: monokaryon.

Categorie 1	Categorie 2	Categorie 3	Categorie 4
Mk 106	Dk 69	Mk 13	Mk 37
Mk 125	Mk 1	Mk 19	Mk 41
Mk 209	Mk 2	Mk 28	Mk 49
Mk 214	Mk 5	Mk 33	Mk 57
Mk 232	Mk 15	Mk 34	Mk 55
Mk 233	Mk 27	Mk 38	Mk 74
	Mk 31	Mk 90	Mk 117
	Mk 32	Mk 100	Mk 124
	Mk 45	Mk 101	Mk 126
	Mk 60	Mk 103	Mk 216
	Mk 70	Mk 113	Mk 229
	Mk 78	Mk 127	
	Mk 82	Mk 201	
	Mk 85	Mk 219	

Mk 88	Mk 225
Mk 93	Mk 234
Mk 96	
Dk 3174	
Mk 107	
Mk 108	
Mk 115	
Mk 119	
Mk 129	
Mk 132	
Mk 203	
Mk 217	
Mk 223	
Mk 226	
Mk 228	

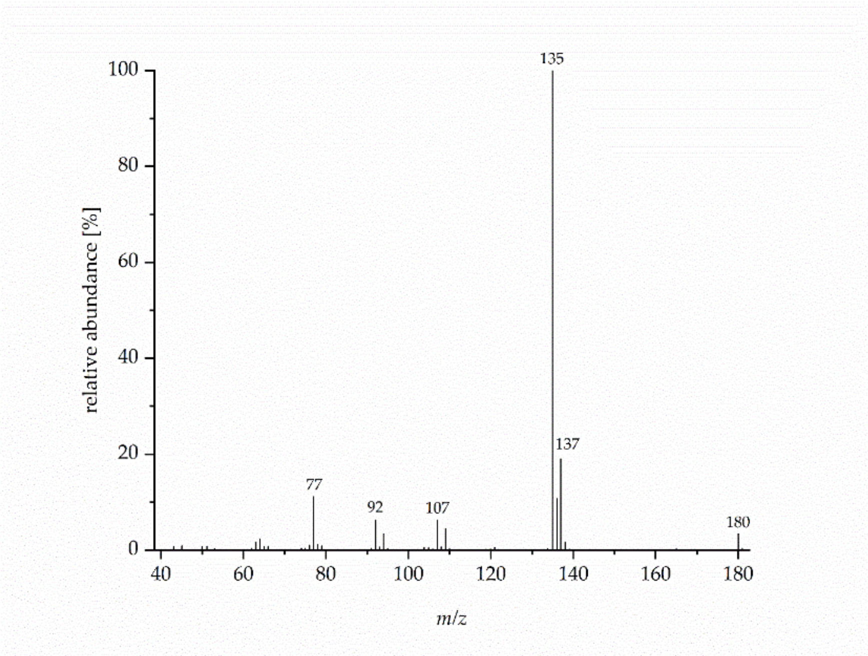

Figure S1 Mass spectrum (EI, 70 eV) of 2-HPP **48**.

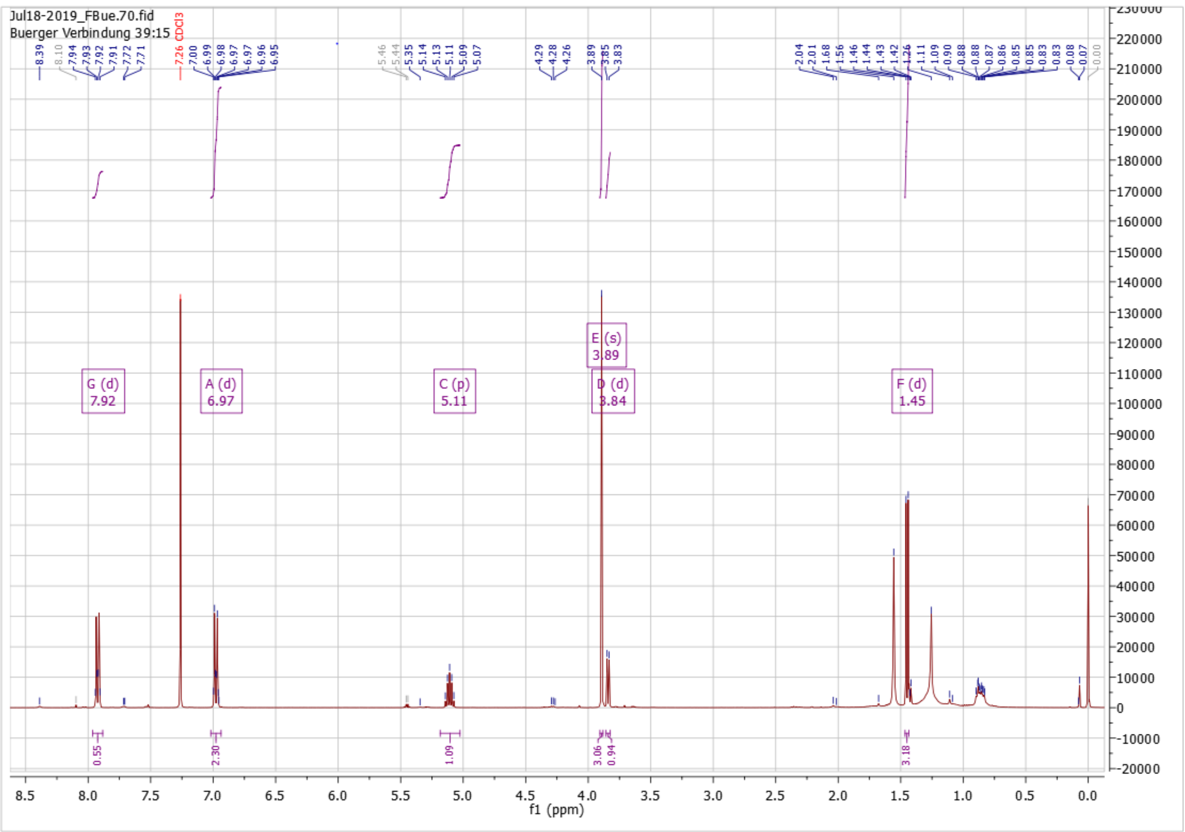

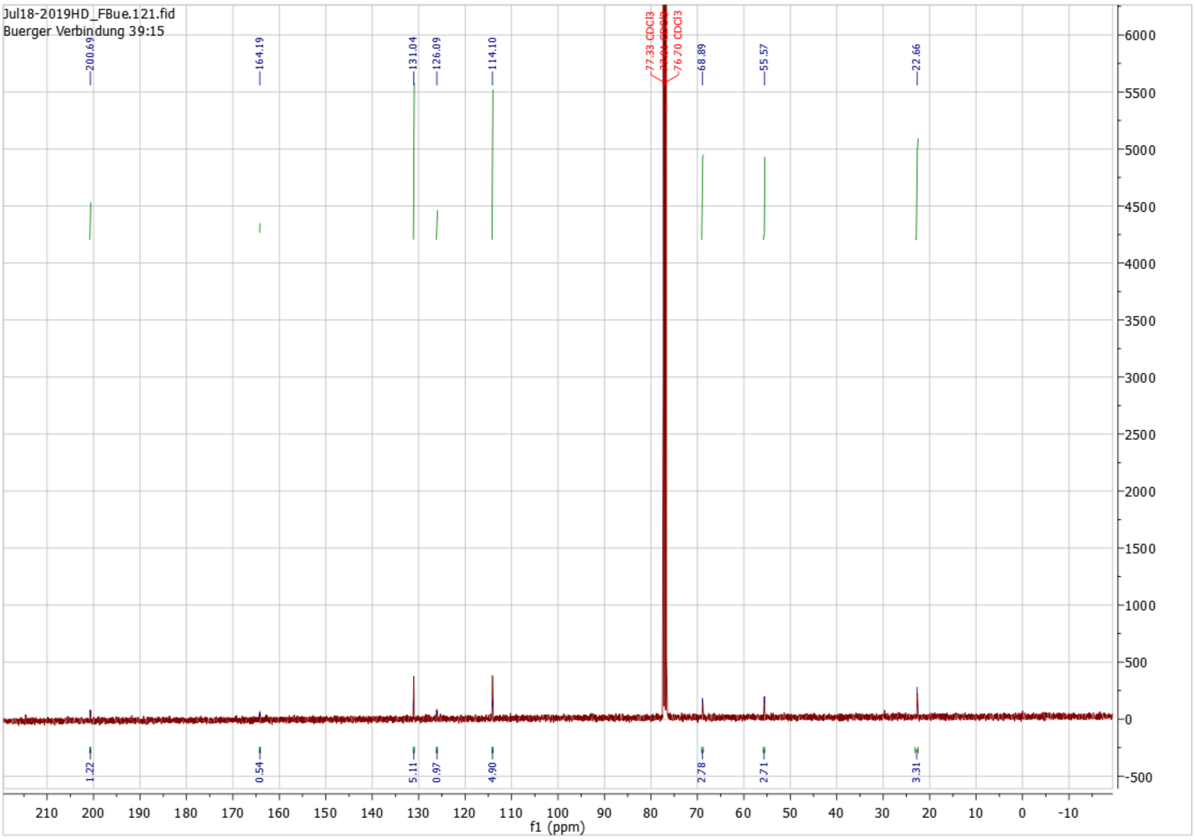

Figure S2 ^{1}H and ^{13}C NMR spectra of 2-HPP **48**.

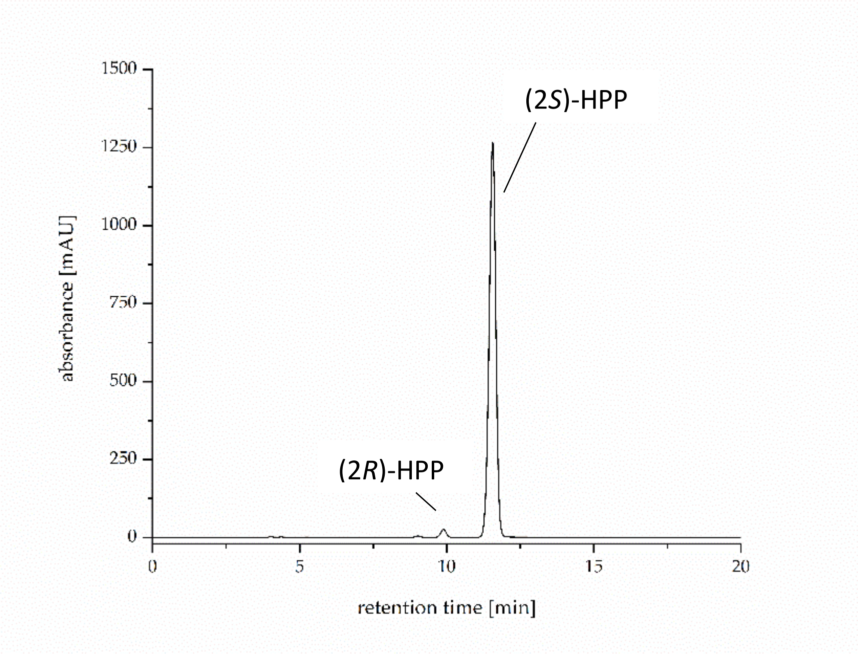

Figure S3 Chromatogram of isolated 2-HPP **48** by chiral HPLC analysis.

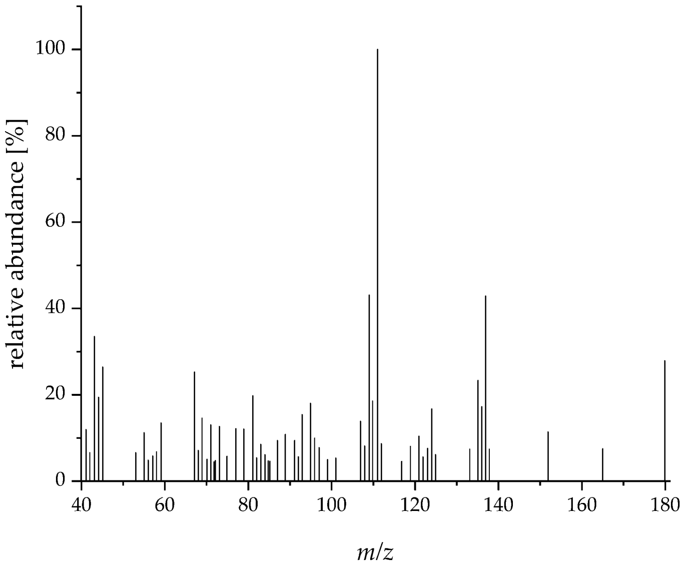

Figure S4 Mass spectrum (EI, 70 eV) of **42**.

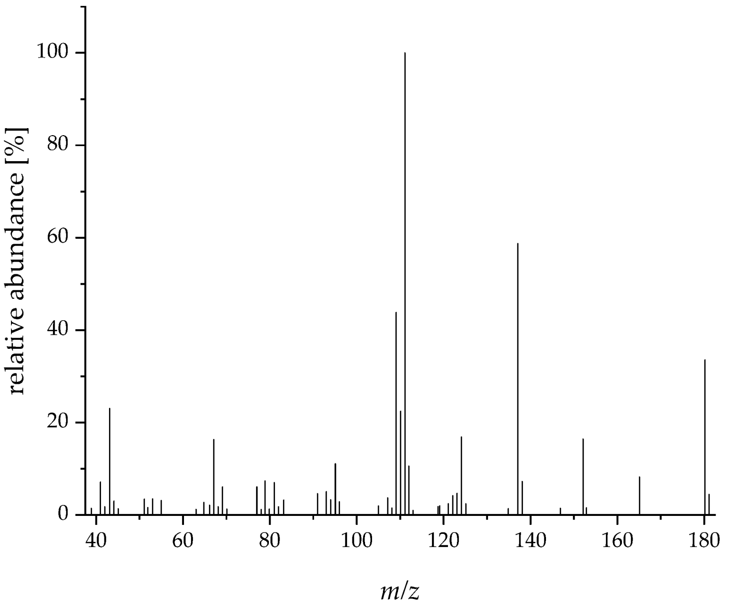

Figure S5 Mass spectrum (EI, 70 eV) of 5,6,7,7a-tetrahydro-4,4,7a-trimethyl-2(4*H*)-benzofuranone.

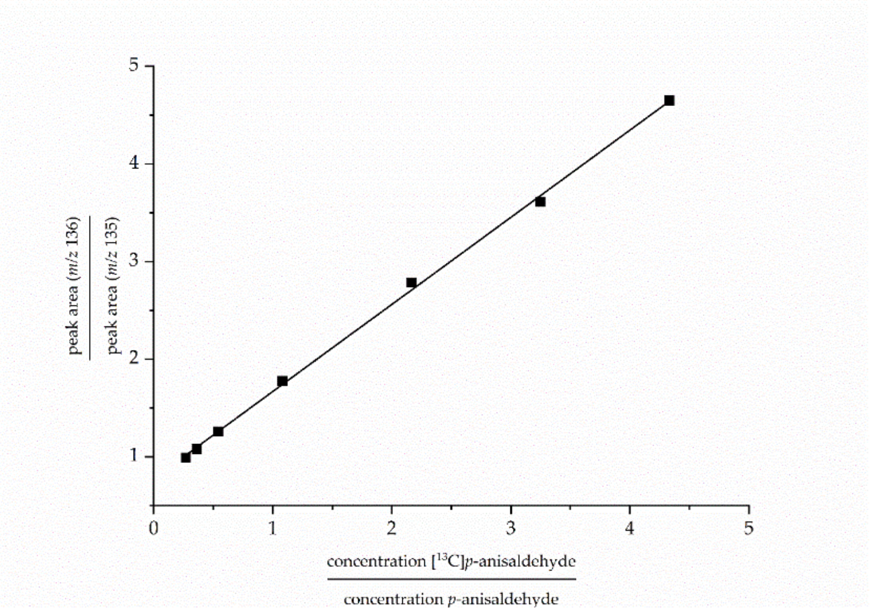

Figure S6 Calibration curve obtained by gas chromatography-mass spectrometry of defined mixtures of *p*-anisaldehyde and [^{13}C]*p*-anisaldehyde.

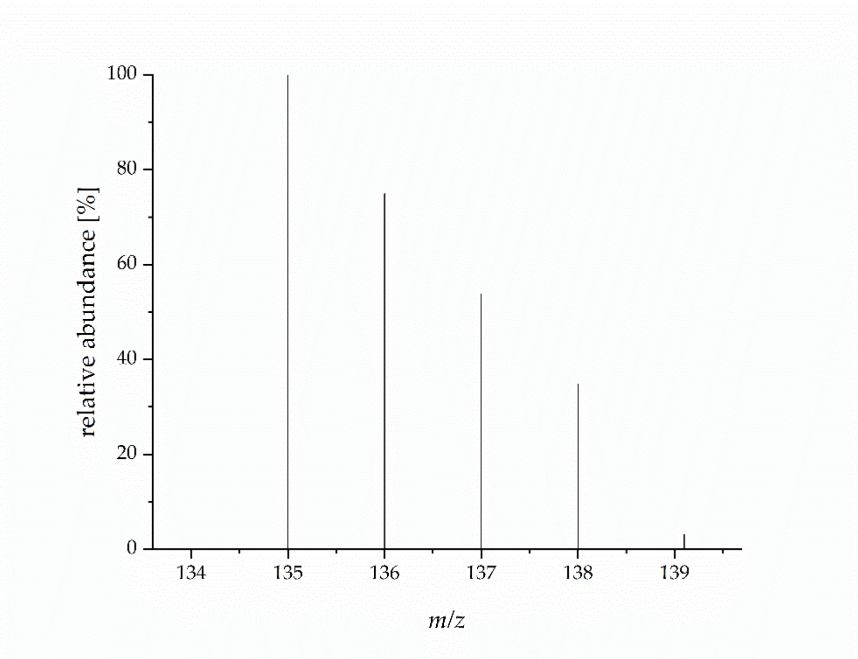

Figure S7 Section of mass spectrum (EI, 70 eV) of [3,5-^{2}H]-4-methoxybenzaldehyde of PSA Dk culture supplemented with L-2-amino-3-([3,5-^{2}H]-4-hydroxyphenyl)-propanoic acid.

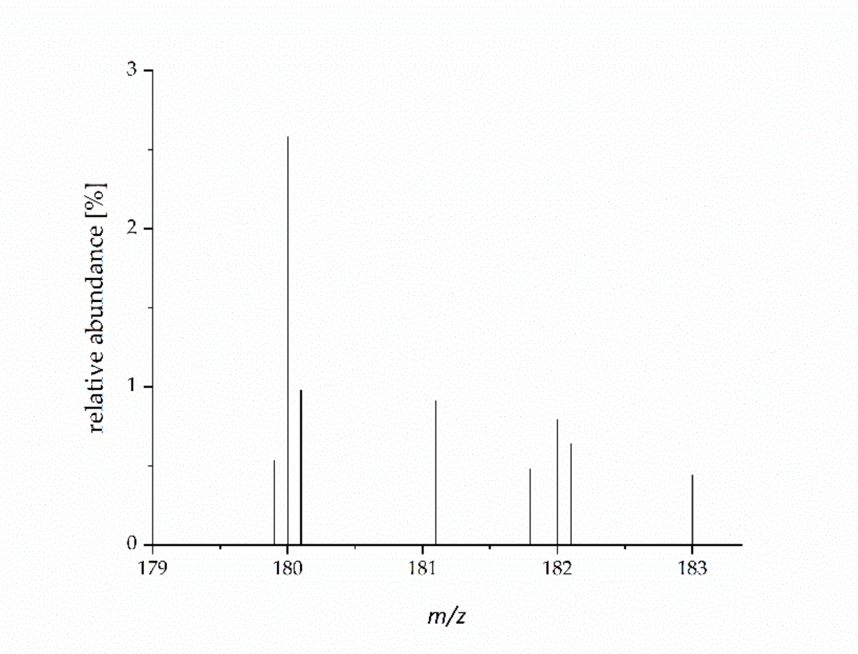

Figure S8 Section of mass spectrum (EI, 70 eV) of 2-hydroxy-1-([3,5-^{2}H]-4-methoxyphenyl)-1-propanone of PSA Dk culture supplemented with L-2-amino-3-([3,5-^{2}H]-4-hydroxyphenyl)-propanoic acid.

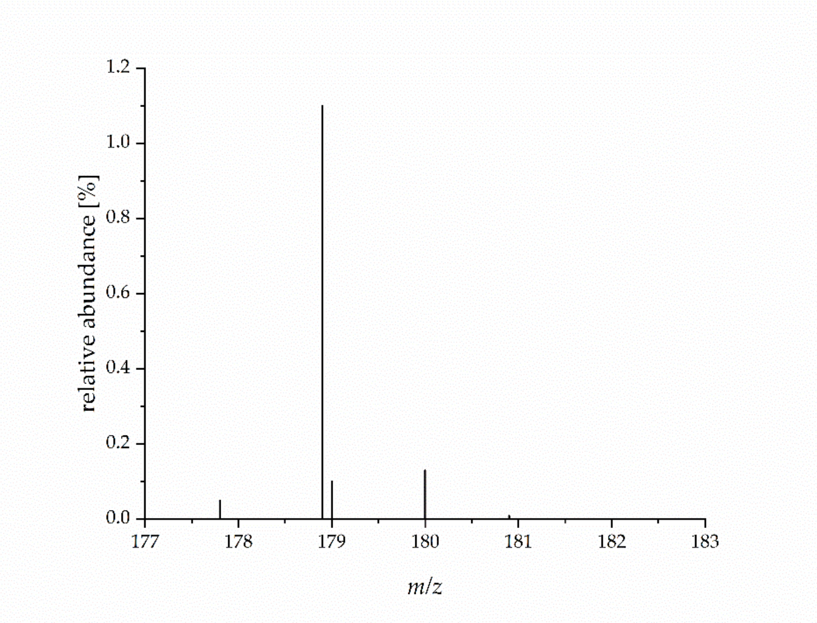

Figure S9 Section of mass spectrum (EI, 70 eV) of 1-(4-methoxyphenyl)propane-1,[2-^{13}C]-dione.

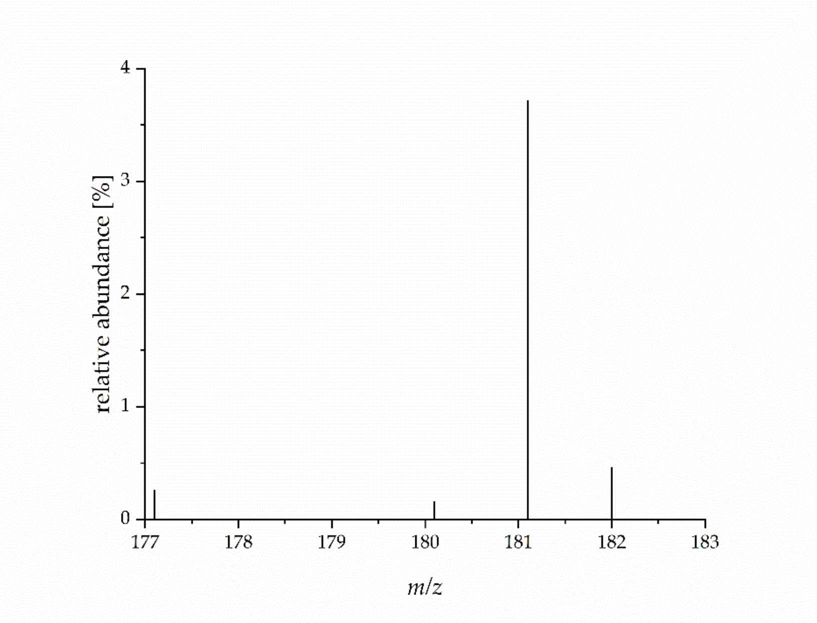

Figure S10 Section of mass spectrum (EI, 70 eV) of [2-^{13}C]-hydroxy-1-(4-methoxyphenyl)-1-propanone after biotransformation of 1-(4-methoxyphenyl)propane-1,[2-^{13}C]-dione by PSA Mk 37 lyophilisate.

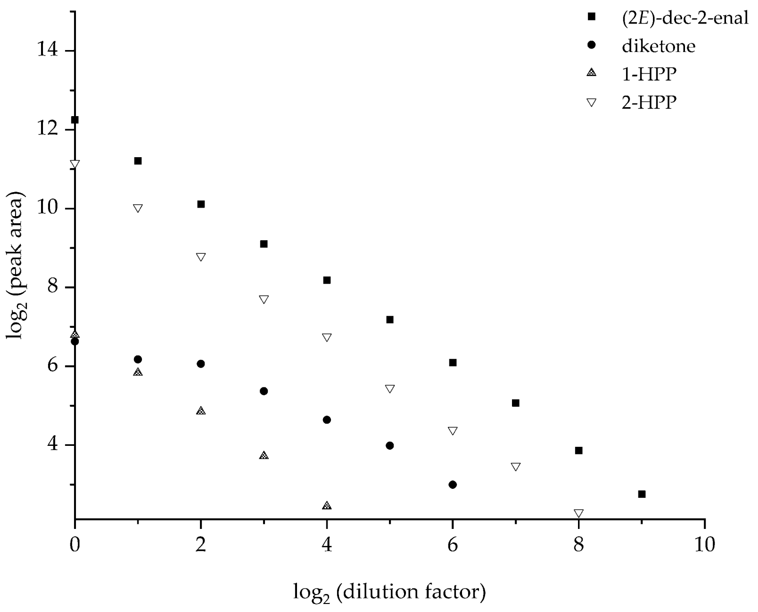

Figure S11 Log2-plotting of the peak areas of detected compounds against the dilution factor, analyzed by gas chromatography-flame ionization detection.
